# Cervical Adenocarcinoma: What's Special About the Long‐Term Reproductive and Oncological Outcomes of Fertility‐Sparing Radical Trachelectomy in It?

**DOI:** 10.1002/cam4.71366

**Published:** 2025-11-08

**Authors:** Jingwen Gan, Dongyan Cao, Huimei Zhou, Mei Yu, Tao Wang, Ying Zhang, Ninghai Cheng, Peng Peng, Jiaxin Yang, Huifang Huang, Keng Shen

**Affiliations:** ^1^ National Clinical Research Center for Obstetric & Gynecologic Diseases, Department of Obstetrics and Gynecology Peking Union Medical College Hospital, Chinese Academy of Medical Sciences & Peking Union Medical College, Peking Union Medical College Hospital (Dongdan Campus) Beijing China

**Keywords:** cervical adenocarcinoma, fertility outcomes, fertility preservation, prognosis outcomes, radical trachelectomy

## Abstract

**Objective:**

To present reproductive and oncological outcomes of radical trachelectomy (RT) in patients with cervical adenocarcinomas (AC).

**Methods:**

This retrospective study included 51 patients with cervical AC who underwent RT at Peking Union Medical Hospital from January 1, 2005 to June 1, 2023.

**Results:**

Five patients (9.8%) experienced cervical stenosis following RT, which likely occurred in cases of abdominal RT (50%) and virginal prophylactic cerclage (33.33%) and those without copper T intrauterine devices during RT (20%). In total, 30 patients (58.82%) attempted to conceive, and 11 (36.67%) succeeded. Five patients (45.45%) achieved pregnancy with fertility assistance. The mean surgery–pregnancy interval was 27 months (range, 17–118). Two preterm and two full‐term births were achieved. With a median follow‐up of 50 months (range, 7–238), seven patients (13.73%) experienced recurrence and three (5.88%) died. Six of seven patients relapsed beyond the residual cervix. The cancer recurrence rate (CRR) was 5.88% for patients with pre‐cervical conization and 17.65% for those with biopsy (*p* = 0.250); 11.63% had human papillomavirus‐associated (HPVA) disease and 25% had non‐HPVA (NHPVA) (*p* = 0.313). The cancer death rate (CDR) was 4.65% with HPVA and 12.50% with NHPVA (*p* = 0.386); 13.63% had the endogenous type and 0 had the exogenous type (*p* = 0.04). Chemotherapy in patients with risk factors resulted in better CRR and CDR than in those without (5.88% vs. 17.65%, 0% vs. 8.82%). The cumulative 5‐year recurrence‐free survival (RFS) and overall survival rates were 82.03% and 94.39%, respectively.

**Conclusion:**

RT in patients with AC led to an acceptable pregnancy rate but a higher CRR and lower 5‐year RFS. Careful patient selection for RT, combined with adjuvant chemotherapy when indicated, is crucial to optimize the balance between reproductive and oncological outcomes in AC.

## 1 Introduction

Adenocarcinoma (AC) accounts for approximately one‐quarter of cervical malignancies, with a significant proportion involving child‐bearing women [1]. Therefore, fertility‐sparing surgery (FSS) has become a priority for women diagnosed with AC who wish to preserve their fertility [2, 3]. However, a great part of AC is located in the cervical canal and is hard to diagnose; moreover, with multifocal extension and “skip lesions” [4], it is more aggressive and prone to paracentral invasion and distant metastasis than squamous cell carcinoma (SCC). Currently, whether the oncological outcomes of patients with AC who undergo FSS are comparable with those with SCC remains controversial [5, 6]. Radical trachelectomy (RT) is the most widely accepted choice for reproductive‐age women with early‐stage cervical cancer [7], but few studies have assessed the oncological and reproductive outcomes of patients with AC undergoing RT. This study aimed to evaluate the feasibility, safety, and fertility outcomes of cervical AC treatment.

## 2 Methods

This retrospective study was conducted at a Grade A tertiary hospital. This study was approved by the Institutional Review Board of Peking Union Medical College Hospital (approval no. K4898). The requirement for informed consent was waived in accordance with the guidelines of the Declaration of Helsinki. We retrospectively analyzed the database of all patients who received fertility‐sparing RT from January 2005 to June 2023 at our institution. The inclusion criteria were: (1) available medical records confirming a histological diagnosis of cervical AC, (2) International Federation of Gynecology and Obstetrics (FIGO) 2018 stage IA1 with lympho‐vascular space invasion (LVSI) or positive surgical margin to IB3 disease (assessed clinically or with magnetic resonance imaging), and (3) having undergone fertility‐sparing RT. The exclusion criteria were: (1) histology of small cell neuroendocrine, serous, or gastric‐type AC; (2) having undergone radical hysterectomy (RH) or radiation therapy after RT owing to positive margins or pelvic lymph node involvement; (3) presence of multiple tumors and cervical cancer not as the primary diagnosis; and (4) detailed medical records were unavailable.

All specimens were independently reviewed by two pathologists, and professional group discussions were consulted if the diagnosis of the specimen remained controversial. Patients were regularly followed up by a gynecological oncologist every 3–4 months in the first 3 years, every 6 months until 5 years, and every year after that. The follow‐up duration was defined from the day of RT surgery to the day of the last follow‐up, death, or being lost to follow‐up. The follow‐up consisted of imaging studies (including pelvic and abdominal ultrasonography or computed tomography imaging and chest radiography), serum tumor markers (e.g., cancer antigen 125), and yearly testing for high‐risk HPV infection and cytology. Recurrence was confirmed through biopsy or positive positron emission tomography–computed tomography scan results.

After a 6‐month follow‐up period with no evidence of recurrent disease, patients were counseled to attempt conception. During follow‐up, reproductive counseling was encouraged, with recommendations for early referral to assisted reproductive technologies if indicated, particularly for patients failing to conceive after 6 months of attempting pregnancy naturally. Upon confirmation of pregnancy (via serum beta‐human chorionic gonadotropin testing and pelvic ultrasound), patients were referred to obstetric clinics for perinatal risk assessment. Obstetricians collaborated with gynecologic oncologists for maternal surveillance during pregnancy and the postpartum period. Cervical cytology screening was regularly performed before and after pregnancy. Any abnormal cytology results prompted comprehensive gynecological evaluation and colposcopy.

Demographic data, characteristics of tumors, key information in treatment, and pathological outcomes were collected. The fertility outcomes included the total number of reproductive attempts, pregnancy rate (PR), number of pregnancies per assisted reproductive technology (ART) and natural attempts, mean surgery–to‐pregnancy interval (SPI), and live birth rate (LBR) among patients who became pregnant. The oncological outcomes included the cancer recurrence rate (CRR), cancer death rate (CDR), recurrence‐free survival (RFS), and overall survival (OS).

In accordance with the journal's guidelines, we will provide our data for independent analysis by a team selected by the Editorial Team for the purposes of additional data analysis or for the reproducibility of this study in other centers if such is requested.

Statistical analysis was performed using SPSS software (version 25.0; Chicago, IL, USA). Continuous variables are expressed as mean ± standard deviation or median (range), and statistical differences were assessed using Student's *t*‐test and the Mann–Whitney *U* test. Categorical variables are reported as numbers (percentages), and statistical differences were assessed using Pearson's chi‐squared test. The Kaplan–Meier method was used to estimate RFS and OS. All hypothesis testing was two‐sided, and *p* < 0.05 was considered statistically significant.

## 3 Results

### 3.1 Characteristics of the Patients

A total of 53 patients received fertility‐sparing RT, while two patients (3.77%) received further RH based on pelvic lymph node metastasis (PLNM) and histology of small cell neuroendocrine postoperative findings, respectively. The median age at diagnosis of the 51 remaining patients was 31 years (range, 22–45). Seventeen patients (33.33%) received histological confirmation through cervical conization and 34 (66.67%) through biopsy. Two patients (3.92%) were at FIGO stage IA1 with LVSI, two (3.92%) were at stage IA2, 40 (78.44%) at stage IB1, six (11.76%) at stage IB2, and one (1.96%) at stage IB3. Forty‐three patients (84.32%) had HPVA, including 11 (21.57%) silva A, seven (13.73%) silva B, and three (5.88%) silva C; 22 (43.14%) had missing information. Eight (15.68%) patients had NHPVA, including three (5.88%) with endometrioid carcinoma of the cervix (ECC) and five (9.80%) with clear cell adenocarcinomas (CCAC). In total, 22 patients (43.14%) had an endogenous tumor, and 29 (56.86%) had an exogenous lesion. Seven patients had a tumor larger than 2 cm, and four of them received NACT (Table 1).

**TABLE 1:** Demographic data and characteristics of tumor before RT.

	Total number (*n* = 51)
Age, years (range)	31 (22–45)
Gravidity, *n* (SD)	0.76 ± 0.91
Parity, *n* (SD)	0.22 ± 0.42
Histology, *n* (%)	
HPVA	43 (84.32%)
Silva A	11 (21.57%)
Silva B	7 (13.73%)
Silva C	3 (5.88%)
Unknown	22 (43.14%)
NHPVA	8 (15.68%)
ECC	3 (5.88%)
CCAC	5 (9.80%)
Tumor growth type, *n* (%)	
Endogenous type	22 (43.14%)
Exogenous type	29 (56.86%)
Preoperative tumor size, *n* (%)	
≤ 20 mm	44 (86.27%)
> 20 mm	7 (13.73%)
FIGO stage, *n* (%)	
IA1 + LVSI	2 (3.92%)
IA2	2 (3.92%)
IB1	40 (78.44%)
IB2	6 (11.76%)
IB3	1 (1.96%)

Abbreviations: CCAC, clear cell adenocarcinoma; CKC, cold knife conization; ECC, endometrioid carcinoma of cervix; HPVA, human papillomavirus‐related adenocarcinoma; NACT, neoadjuvant chemotherapy; NHPVA, non‐human papillomavirus‐related adenocarcinoma.

### 3.2 Surgical and Pathological Data of the Patients

Forty‐five patients (88.24%) received minimally invasive (MIS) RT, including 15 modified laparoscopic RT (LRT), 18 traditional LRT, and 12 vaginal RT (VRT); the other six (11.76%) received abdominal RT (AbRT). Virginal prophylactic cerclage (VPC) was conducted in six patients (11.76%), and a copper T intrauterine device (IUD) was inserted in 36 patients (70.59%) during RT to prevent endocervical obstruction. Five patients (9.8%) had cervical stenosis and abnormal uterine bleeding, which were treated with cervical dilatation; one experienced vaginal cuff dehiscence twice and received surgical repair (Table 2). Of five patients with cervical stenosis, three received AbRT (3/6, 50%), and the other two underwent MIS RT (2/45, 4.44%); two of them had VPC (2/6, 33.33%) while the other did not (3/45, 6.67%); two patients had copper T IUDs (2/36, 5.56%) while the other had not (3/15, 20%). AbRT and VPC significantly increased the risk of cervical stenosis (*p* < 0.001 and *p* = 0.039, respectively), while the copper T IUD clinically reduced the risk (*p* > 0.05). Seven patients (13.73%) received platinum‐based postoperative adjuvant chemotherapy (PACT) owing to deep stromal infiltration (DSI), LVSI, or positive margins (Table 2). Two patients had positive resection margins but refused to undergo further RH or radiotherapy. One patient at FIGO stage IB1 with the adenocarcinoma identified < 1 mm in 1 and 2° area of the incisal margin of the resection specimen chose to follow up without further treatment; another one at FIGO stage IB1 with adenocarcinoma at the incisal margin of the resection cervix received three cycles of paclitaxel and carboplatin adjuvant chemotherapy after RT instead of RH.

**TABLE 2:** Details of treatment and pathological data.

	Total number (*n* = 51)
Diagnostic method, *n* (%)	
Biopsy	34 (66.67%)
CKC	17 (33.33%)
NACT, *n* (%)	
Y	4 (7.84%)
N	47 (92.16%)
Surgical procedure, *n* (%)	
LRT	33 (64.71%)
AbRT	6 (11.76%)
VRT	12 (23.53%)
Cervical cerclage, *n* (%)	8 (15.69%)
During RT surgery by virgin	6 (11.76%)
During first trimester by laparoscopy	2 (3.93%)
Copper T IUD	
N	15 (29.41%)
Y	36 (70.59%)
Postoperative complications, *n* (%)	6 (11.76%)
Cervical stenosis	5 (9.8%)
Vaginal cuff dehiscence	1 (1.96%)
PLNM, *n* (%)	0
Parametrial infiltration, *n* (%)	0
Margin, *n* (%)	
Y	2 (3.92%)
N	49 (96.08%)
LVSI, *n* (%)	
Y	5 (9.80%)
N	46 (90.20%)
Stromal infiltration, *n* (%)	
Superficial	46 (90.20%)
Deep	5 (9.80%)
PACT, *n* (%)	
Y	7 (13.73%)
N	44 (86.27%)

Abbreviations: AbRT, abdominal radical trachelectomy; IUD, intrauterine device; LRT, laparoscopic radical trachelectomy; LVSI, Lympho‐vascular stromal invasion; PACT, postoperative adjuvant chemotherapy.; PLNM, pelvic lymph node metastasis; VRT, vaginal radical trachelectomy.

### 3.3 Fertility Outcomes of the Patients

Thirty patients (58.82%) attempted conception, among them, 11 (36.67%) achieved a total of 11 pregnancies after a median interval of 27 months (range, 17–118), resulting in four live births (36.36% of pregnancies), all delivered by cesarean section. Pregnancy was spontaneously achieved in six patients (54.55%), whereas five patients used in vitro fertilization and embryo transfer (IVF‐ET) (45.45%). None of the five patients with cervical stenosis achieved successful pregnancy even after cervical dilatation. There were two full‐term births at 37 weeks, including one with laparoscopic prophylactic cerclage (LPC) at 11 weeks after pregnancy and another without intervention before CS. Among two preterm births, one patient undergoing VPC experienced preterm birth at 26 weeks due to intrauterine infection and another receiving LPC terminated the pregnancy at 36 weeks because of regular uterine contractions and a history of CS. Among the patients without live births, two terminated their pregnancies in the first trimester owing to embryo arrest, one had a late miscarriage at 22 weeks because of cervical incompetence, two underwent artificial abortion for personal reasons, one experienced cornual pregnancy and received unilateral salpingectomy, and one patient had failed embryo implantation after elevated serum human chorionic gonadotropin was detected (Tables 3 and 4).

**TABLE 3:** Reproductive and oncological outcomes.

Total number (*n* = 51)	
Attempt to conceive, *n* (%)	30 (58.82%)
Method of attempt to conceive, *n* (%)	
SC	19 (63.33%)
IVF‐ET directly	3 (10%)
SC turn to ART	8 (26.67%)
Total pregnancy rate, *n* (%)	11 (36.67%)
By SC	6 (54.55%)
By IVF‐ET	5 (45.45%)
SPI, month (range)	27 (17–118)
Pregnancy outcomes, *n* (%)	
Preterm deliveries	2 (18.18%)
Full‐term deliveries	2 (18.18%)
Embryo arrest	2 (18.18%)
Artificial abortion	2 (18.18%)
Late miscarriage	1 (9.09%)
Cornual pregnancy	1 (9.09%)
Failed embryo implantation	1 (9.09%)
Median follow‐up time, month (range)	50 (7–238)
Recurrence, *n* (%)	7 (13.73%)
Time to recurrence, month (range)	22 (8–62)
Death of disease, *n* (%)	3 (5.88%)

Abbreviations: ART, assisted reproductive technology; IVF‐ET, in vitro fertilization and embryo transfer; SC, spontaneous conception; SPI, surgery‐to‐pregnancy interval.

**TABLE 4:** Description of cases with pregnancies.

Case	Age	Histology	Tumor size	FIGO stage	Surgical procedure	Cerclage	Method of conception	SPI (months)	Pregnancy outcome	Recurrence	Death
1	25	HPVA	11	IB1	AbRT	VPC	IVF‐ET	33	Embryonic demise at 10 weeks of gestation	NR	N
2	28	HPVA	10	IB1	VRT	N	SC	27	Artificial abortion at the first trimester	R (Brain)	Y
3	31	CCAC	18	IB1	VRT	LPC	SC	101	36‐week preterm birth	NR	N
4	38	HPVA	19	IB1	LRT	N	IVF‐ET	34	Biochemical pregnancy	NR	N
5	34	HPVA	16	IB1	LRT	N	SC	27	Artificial abortion at the first trimester	NR	N
6	31	HPVA	20	IB1	LRT	N	SC	26	EP	NR	N
7	34	HPVA	12	IB1	LRT	VPC	IVF‐ET	39	Embryonic demise at 8 weeks of gestation	NR	N
8	30	HPVA	35	IB2	NACT+LRT	LPC	SC	17	37‐week full‐term birth	NR	N
9	37	CCAC	0.2	IA1 + LVSI	LRT	VPC	IVF‐ET	118	26‐week preterm birth	NR	N
10	27	HPVA	0.4	IA2	LRT	N	IVF‐ET	59	37‐week full‐term birth	NR	N
11	31	HPVA	Positive margin after CKC	IB1	LRT	N	SC	17	Late miscarriage at 22 weeks of gestation	NR	N

Abbreviations: AbRT, abdominal radical trachelectomy; CCAC, clear cell adenocarcinoma; CKC, cold knife conization; EP, ectopic pregnancy; HPVA, human papillomavirus‐related adenocarcinoma; IVF‐ET, in vitro fertilization and embryo transfer; LRT, laparoscopic radical trachelectomy; NACT, neoadjuvant chemotherapy; NR, no recurrence; R, recurrence; SC, spontaneous conception; SPI, surgery‐to‐pregnancy interval; VRT, vaginal radical trachelectomy.

### 3.4 Oncologic Outcomes of the Patients

With a median follow‐up of 50 months (range, 7–238), seven patients (13.73%) experienced recurrences. Among them, four (57.14%) experienced recurrence within 2 years of treatment and six (85.71%) within 5 years. The median duration between primary RT surgery and recurrence was 22 months (range, 8–62). Three patients (5.88%) died during follow‐up. The cumulative 5‐year RFS and OS rates were 82.03% and 94.39%, respectively. Among these seven patients, one relapsed only in the cervical stump; four in the pelvis, including the parametrial tissue, uterine corpus and rectum, adnexa, and pelvic LN; and two had tumor metastasis in distant sites (brain and Virchow LN respectively) (Tables 3 and 5).

**TABLE 5:** Information of recurrence cases.

Case	Histology	FIGO stage	Endo−/Exo	Pre‐CKC	Risk factors	CT	Surgical procedure	DFI (months)	Recurrent sites	Treatment	Follow‐up time	Outcome
1	HPVA	IB1	Exo	N	N	N	VRT	8	P (parametrial and right adnexa mass)	S + CCRT	158	NED
2	HPVA	IB1	Exo	N	N	N	AbRT	9	CP + P (cervical stump involved virgin, uterine corpus and sigmoid)	CCRT + CT	38	NED
3	ECC	IB1	Endo	N	N	N	VRT	10	CP + EP (cervical stump + Virchow LN)	S + CCRT + CT	31	DOD
4	HPVA	IB1	Exo	N	N	N	VRT	22	CP (cervical stump)	S	131	NED
5	HPVA	IB1	Endo	Y	N	N	VRT	28	EP (Brain)	CT	46	DOD
6	HPVA	IB1	Endo	N	N	N	LRT	33	P (multiple pelvic mass + pelvic LN)	S + CCRT + CT	66	DOD
7	ECC	IB2	Exo	N	3 cm	Y	VRT	62	CP + P (cervical stump + pelvic LN)	S + RT	238	NED

*Note:* Risk factors included tumor over 2 cm, CCAC, LVSI, DSI or positive surgical margin.

Abbreviations: AbRT, abdominal radical trachelectomy; CP, central pelvis; CT, chemotherapy; DOD, dead of disease; DSI, deep stromal infiltration; ECC, endometrioid carcinoma of cervix; EP, extra pelvis; LN, lymph node; LRT, laparoscopic radical trachelectomy; LVSI, lympho‐vascular stromal invasion; NED, no evidence of disease; P, pelvic; PACT, postoperative adjuvant chemotherapy; Pre‐CKC, preoperative cervical conization; S, surgery; VRT, vaginal radical trachelectomy.

The CRR was 5.88% for patients with preoperative cervical conization and 17.65% for those with biopsy (*p* = 0.250), 11.63% for those with HPVA and 25% for those with NHPVA (*p* = 0.313), 14.29% for those with tumors larger than 2 cm and 13.64% for those with tumors ≤ 2 cm (*p* = 0.963), 13.64% for those with the endogenous type and 13.79% for those with the exogenous type (*p* = 0.987), 13.33% for those with MIS RT and 16.67% for those with AbRT (*p* = 0.823). Nine in 17 patients with risk factors including tumors larger than 2 cm, histology of CCAC, LVSI, DSI, or positive surgical margins received chemotherapy (52.94%) and one relapsed (5.88%), whereas the recurrence rate in those without risk factors was 17.65% (*p* = 0.250) (Table 6).

**TABLE 6:** Risk factors of recurrence and death.

	No recurrence (*n* = 44)	Recurrence (*n* = 7)	*χ* ^2^	*p*	No death (*n* = 48)	Death (*n* = 3)	*χ* ^2^	*p*
Pre‐CKC								
N	28 (82.35%)	6 (17.65%)	1.325	0.250	32 (94.12%)	2 (5.88%)	/	/
Y	16 (94.12%)	1 (5.88%)			16 (94.12%)	1 (5.88%)		
Histology								
HPVA	38 (88.37%)	5 (11.63%)	1.019	0.313	41 (95.35%)	2 (4.65%)	0.751	0.386
NHPVA	6 (75%)	2 (25%)			7 (87.50%)	1 (12.50%)		
Tumor size								
≤ 2 cm	38 (86.36%)	6 (13.64%)	0.002	0.963	41 (93.18%)	3 (6.81%)	0.507	0.476
> 2 cm	6 (85.71%)	1 (14.29%)			7 (100%)	0		
Growth pattern								
Endogenous	19 (86.36%)	3 (13.64%)	0	0.987	19 (86.36%)	3 (13.64%)	4.201	0.040*
Exogenous	25 (86.21%)	4 (13.79%)			29 (100%)	0		
Surgical approach								
MIS RT	39 (86.67%)	6 (13.33%)	0.050	0.823	42 (93.33%)	3 (6.67%)	0.425	0.514
AbRT	5 (83.33%)	1 (16.67%)			6 (100%)	0		
Risk factors								
N	28 (82.35%)	6 (17.65%)	1.324	0.250	31 (91.18%)	3 (8.82%)	1.594	0.206
Y	16 (94.12%)	1 (5.88%)			17 (100%)	0 (5.88%)		

*Note:* Risk factors included tumor over 2 cm, CCAC, LVSI, DSI or positive surgical margin.

Abbreviations: MIS, minimally invasive; Pre‐CKC, preoperative cervical conization.

* *p* < 0.05.

The CDR for patients with preoperative cervical conization was the same as that for those with biopsy (5.88%), 4.65% for those with HPVA and 12.50% for those with NHPVA (*p* = 0.386), 0 for those with tumors larger than 2 cm and 6.82% for those with tumors ≤ 2 cm (*p* = 0.476), 13.63% for those with the endogenous type and 0 for those with the exogenous type (*p* = 0.04), 6.67% for those with MIS RT and 0 for those with AbRT (*p* = 0.514). None of the patients with risk factors died during follow‐up, while the CDR of those without risk factors was 8.82% (*p* = 0.206) (Table 6).

## 4 Discussion

Previous studies have reported favorable oncologic outcomes and reproductive potential for patients with cervical cancer undergoing RT irrespective of histology [8, 9, 10]. However, data specifically addressing AC remain limited. In this analysis of patients with AC managed with RT, we observed that approximately 60% of women actively attempted to conceive after RT surgery. Among these, the rates of successful pregnancy and live birth were 36.67% and 36.36%, respectively. Notably, nearly half of the pregnancies (45.45%) were achieved through ART, primarily IVF‐ET, while only 10% of patients directly opted for ART after RT. The CRR and CDR in our study were 13.73% and 5.88%, respectively. Most recurrences (85.71%) occurred outside the residual cervix (pelvic or distant sites). Factors potentially associated with improved prognosis included preoperative cervical conization, HPVA histology, exogenous tumor growth pattern, AbRT, and administration of chemotherapy to patients with identified risk factors. The cumulative 5‐year RFS rate and OS rates were 82.03% and 94.39%, respectively.

Previous studies have analyzed the rate of fertility preservation in patients undergoing RT by excluding those who required further RH or adjuvant therapy, indicating that fertility preservation was achieved in 69.3%–100% of patients undergoing AbRT, with an overall rate of 85% across histological types and 91.1% among patients who underwent VRT [11, 12]. Patients receiving adjuvant chemotherapy were included in our calculation of fertility preservation outcomes. Four patients (7.55%) with high‐risk characteristics (specific unfavorable histology, PLNM, and/or positive margins) were recommended RH post‐RT, while 51 patients (96.2%) ultimately retained their fertility. This fertility preservation rate was consistent with that observed in our previously reported cervical cancer cohort with high‐risk characteristics managed with concurrent chemoradiotherapy or RH after RT (3/153, 2.0%) [8], and no worse than the rate specifically reported for patients with SCC (3/43, 6.9%) [13]. These findings suggest that RT is feasible for AC as a fertility‐sparing surgery.

In previous literature, pregnancy outcomes have been highly variable. The PRs were 49.4%–67.5% following VRT, with LBRs of 63.4%–65.0%. For AbRT, PRs ranged from 41.9%–43.2% and LBRs were 63.4%–65.7%. Following LRT, PRs were 36.2%–51.5% and LBRs were 56.5%–57.1%. The preterm delivery rate ranged between 20% and 100% [6, 14, 15, 16, 17]. Although the proportion of patients in our study attempting conception (58.82%) was higher than that in some other reports (e.g., 40% [18]), the rates of successful pregnancy and live birth did not demonstrate a superior trend. This discrepancy may be partly explained by the high proportion of nulliparous women (nearly 80%) in our cohort and the absence of formal preoperative fertility assessment. Nevertheless, the PR among patients with AC (36.7%) in this study was relatively higher than that in our previously published cohort of patients with cervical cancer (26.0%) and the existing cohort of patients with SCC (27.8%); a similar trend was also noted in the LBR (36.4% vs. 16.9% and 20%, respectively).

Cervical stenosis, a common complication following RT, adversely affects fertility. Reported stenosis rates in the literature vary per surgical approach: 4.1%–33.3% for VRT, 3.5%–28.0% for AbRT, and 2.0%–50.0% for LRT [6]; our observed rate of 9.8% was near the average (10%). Consistent with prior findings [19, 20], both AbRT and VPC during RT were associated with an increased risk of cervical stenosis in our cohort. Furthermore, VPC has been linked to adverse obstetric outcomes such as pre‐labor rupture of membranes and chorioamnionitis [21, 22]. In contrast, the two patients in our study who underwent LPC during the first trimester experienced no adverse obstetric events. Existing evidence suggests that LPC might better prevent mid‐trimester pregnancy loss and preterm birth than VPC, improving pregnancy outcomes [23, 24]. Therefore, selecting LPC over VPC might represent a superior strategy for balancing residual cervical function against the risk of obstruction. Although not statistically significant in our analysis, the insertion of a copper T IUD during RT appeared to reduce the incidence of complications, supporting its continued recommendation.

Only 10% of our patients directly chose IVF‐ET after RT; however, nearly half of those who achieved pregnancy (45.45%) ultimately required IVF‐ET, often after prolonged (> 1 year) unsuccessful attempts at spontaneous conception. This highlighted a potential gap, as few women appeared to receive comprehensive reproductive counseling before and after RT [25]. Given that the primary objective of FSS is to preserve reproductive potential, facilitating timely conception and optimizing obstetric outcomes is paramount [26], especially considering the potentially higher CRR and shorter RFS observed in AC compared to SCC [5]. Proactive reproductive counseling can empower patients by elucidating the potential impact of RT on future fertility, pregnancy complications, and oncologic surveillance. It also enables the assessment of individual reproductive potential, guiding decisions on the necessity and timing of AbRT intervention [27], thereby potentially expediting fertility treatment and mitigating age‐related pregnancy risks.

Reported CRRs for early‐stage cervical cancer (all histology) after RT range 0%–25%, with CDRs of 0%–7.6% [6, 28]. In published cohorts comprising exclusively or predominantly SCC, the average rates of recurrence and mortality summarized across different surgical approaches showed minimal variation: the average CRR following VRT ranged from 3.7% to 4%, and the average CDR ranged from 1.1% to 1.7%. For AbRT, the average CRR was 3.6% to 3.9%, and the average CDR was 0.7% to 1.4%. Following LRT, the average CRR ranged from 3.3% to 4.2%, and the average CDR ranged from 0.1% to 0.7%. Our study found that the CRR and CDR after RT for AC (13.7% and 5.88%, respectively) not only exceeded the average values reported in the aforementioned literature but were also higher than those (CRR: 5.6%, CDR: 1.6%) reported for our previously published cohort of patients with cervical cancer (SCC, *n* = 135; AC, *n* = 15) [13, 28]. Notably, VRT was used in more than 80% of our recurrent cases. Other studies also noted a tendency for higher recurrence in patients with AC who received RT, particularly following VRT, involving less extensive cervical tissue removal [5, 29]. Consistent with our findings, Zusterzeel et al. [30] observed a CRR of 12.5% in patients with AC treated with VRT compared to 4.2% in corresponding cases of SCC. Given that AC differs from SCC in epidemiology, biology, response to therapy, and recurrence patterns, its surgical management strategies require separate evaluation to prevent a poor prognosis [31]. Besides, reviewing published RT series, researchers observed a trend toward cervical recurrence in patients with AC undergoing VRT, which appeared more pronounced than in those undergoing AbRT (32.3% vs. 12.5%) [5]. In our study, only one recurrence was isolated to the cervical stump, while the remaining six involved pelvic structures or distant sites. Whether this recurrence pattern in patients with AC is related to the surgical approach requires further investigation.

Previous research has identified prognostic factors for early cervical cancer after RT or RH. In our study, patients undergoing preoperative cervical conization exhibited a lower CRR than those diagnosed with biopsy alone. Conization may improve survival by reducing the tumor burden and enabling more precise staging [32]. Additionally, NHPVA demonstrated a trend toward higher CRR and CDR, likely owing to its more aggressive biological behavior [33]. Corrado et al. [34] analyzed the recurrence patterns and oncologic outcomes of 360 patients with stage IB1–IB2 cervical cancer who underwent MIS or abdominal RH, and found no significant difference between the two surgical approaches. Zhu et al. [35] reported similar findings for patients with stage IB1–IIA AC undergoing RH. However, we observed a significantly higher CDR among patients with endogenous lesions and those undergoing MIS RT, echoing the findings of other studies [8, 36, 37]. Therefore, prognostic factor assessment and tailored treatment plans are necessary for each patient diagnosed with AC undergoing RT, emphasizing the critical need for more dedicated studies on AC.

The integration of chemotherapy with RT has led some clinicians to extend RT eligibility to patients with tumors > 2 cm [38]. A meta‐analysis including 139 patients with cervical cancer larger than 2 cm who underwent RT with/without NACT reported 22 pregnancies and 12 live births [39]. RT with/without NACT may comprise a viable option for patients with larger tumors to preserve fertility, while the optimal management, particularly using NACT followed by MIS RT, remains debated. Some researchers have suggested that both NACT combined with MIS RT and primary AbRT are feasible and safe options for selected patients with cervical tumors larger than 2 cm or other risk factors (e.g., high‐risk histologies) [15, 40, 41], whereas some have reported potentially superior CRR and CDR with AbRT than with NACT followed by VRT [42]. In our cohort, four patients with tumors > 2 cm received NACT prior to MIS RT, and three underwent AbRT. No recurrence occurred in the ART group, while one patient with FIGO stage IB2 relapsed after NACT and VRT. Crucially, CRR and CDR were lower for patients with identified risk factors, who received chemotherapy (pre‐ and/or post‐operatively), than for those without risk factors. This suggested that adjuvant chemotherapy might confer a survival benefit for patients with AC with risk factors undergoing RT.

Understanding the distinct patterns of recurrence in cervical adenocarcinoma is crucial for tailoring surveillance and therapeutic strategies. Unlike SCC, adenocarcinoma is often associated with multifocal extension and “skip lesions,” which may contribute to a higher propensity for pelvic and distant recurrence, as observed in our cohort where most recurrences occurred beyond the residual cervix [4]. A comprehensive analysis of prognostic factors, including histologic subtype (HPVA vs. NHPVA), tumor growth pattern, and surgical approach, is essential for risk stratification. Recent evidence underscored the need for a personalized treatment paradigm that integrated these factors to optimize oncologic outcomes while preserving fertility where possible [34]. Tailoring the extent of surgery and the use of adjuvant therapies based on individualized risk assessment could help mitigate recurrence risks in this patient population.

The lymph node status remains one of the most significant prognostic factors for patients with cervical cancer. In the context of fertility‐sparing surgery, where quality of life is paramount, the surgical approach to lymph node assessment is of critical importance. While pelvic lymphadenectomy has been the standard, it carries a non‐negligible risk of postoperative complications such as lympho‐cyst formation and lower limb lymphedema. Sentinel lymph node mapping (SNM) identification has emerged as a highly accurate and minimally invasive alternative for assessing PLNM. Studies have demonstrated that SNM not only reduces the incidence of early lymph‐related complications but also decreases early morbidity and improves quality of life [43, 44, 45]. Specifically, in the context of fertility‐preserving approaches, recent evidence confirms the feasibility and oncological safety of SNM, supporting its role in tailoring surgical management to optimize both oncologic and reproductive outcomes [45]. The ongoing SENTINEL III trial will further elucidate the role of SNM in early cervical cancer [46].

This study was one of the largest series focusing specifically on AC treated with RT and reported reproductive and oncological outcomes with the longest follow‐up duration to date. However, there were limitations inherent to the retrospective design and relatively modest sample size, which might have limited the power to detect significant differences for some variables. Additionally, the strategy of NACT followed by MIS RT requires further validation through larger prospective studies. Several lines of evidence support the potential of specific biomarkers (such as cancer antigen 125, soluble form of interleukin 2 receptor, and tumor necrosis factor for identifying early‐stage cervical cancer and providing patients with a better prognosis) [31]. Further research is needed to advance the clinical translation of these hot topics.

In conclusion, RT remains a viable fertility‐sparing option for selected patients with AC. Employing LPC instead of VPC and placing a copper T IUD during RT may optimize residual cervical function and reduce the stenosis risk. Proactive reproductive counseling and timely access to fertility treatments are critical for achieving successful conception. Patient selection and surgical approach require careful consideration, particularly for those with NHPVA histology or endogenous tumor growth patterns, which may portend a worse prognosis. The administration of adjuvant chemotherapy to patients with high‐risk characteristics (tumor size > 2 cm, CCAC, LVSI, or DSI) appears beneficial and is recommended to improve oncologic outcomes.

## Author Contributions

**Jingwen Gan:** data curation, formal analysis, writing – original draft, software. **Dongyan Cao:** writing – review and editing, funding acquisition, supervision, resources, methodology. **Huimei Zhou:** resources. **Mei Yu:** resources. **Tao Wang:** resources. **Ying Zhang:** resources. **Ninghai Cheng:** resources. **Peng Peng:** resources. **Jiaxin Yang:** resources. **Huifang Huang:** resources. **Keng Shen:** resources.

## Ethics Statement

This study involves human participants and was approved by the Institutional Review Board of Peking Union Medical College Hospital (No. K4898). The requirement for informed consent was waived in accordance with the Declaration of Helsinki.

## Conflicts of Interest

The authors declare no conflicts of interest.

## Data Availability

The data that support the findings of this study are available on request from the corresponding author. The data are not publicly available due to privacy or ethical restrictions.
